# Identification of Enhanced Vaccine Mimotopes for the p15E Murine Cancer Antigen

**DOI:** 10.1158/2767-9764.CRC-23-0384

**Published:** 2024-04-02

**Authors:** Shiqi Zhou, Yiting Song, Yuan Luo, Breandan Quinn, Yang Jiao, Mark D. Long, Scott I. Abrams, Jonathan F. Lovell

**Affiliations:** 1 Department of Biomedical Engineering, State University of New York at Buffalo, Buffalo, New York.; 2 Department of Biostatistics and Bioinformatics, Roswell Park Comprehensive Cancer Center, Buffalo, New York.; 3 Department of Immunology, Roswell Park Comprehensive Cancer Center, Buffalo, New York.

## Abstract

Mimotopes of short CD8^+^ T-cell epitopes generally comprise one or more mutated residues, and can increase the immunogenicity and function of peptide cancer vaccines. We recently developed a two-step approach to generate enhanced mimotopes using positional peptide microlibraries and herein applied this strategy to the broadly used H-2K^b^–restricted murine leukemia p15E tumor rejection epitope. The wild-type p15E epitope (sequence: KSPWFTTL) was poorly immunogenic in mice, even when combined with a potent peptide nanoparticle vaccine system and did not delay p15E-expressing MC38 tumor growth. Following positional microlibrary functional screening of over 150 mimotope candidates, two were identified, both with mutations at residue 3 (p15E-P3C; “3C,” and p15E-P3M; “3M”) that better induced p15E-specific CD8^+^ T cells and led to tumor rejection. Although 3M was more immunogenic, 3C effectively delayed tumor growth in a therapeutic setting relative to the wild-type p15E. As 3C had less H-2K^b^ affinity relative to both p15E and 3M, 15 additional mimotope candidates (all that incorporated the 3C mutation) were assessed that maintained or improved predicted MHC-I affinity. Valine substitution at position 2 (3C2V, sequence: KVCWFTTL) led to improved p15E-specific immunogenicity, tumor rejection, and subsequent long-term antitumor immunity. 3C, 3M, and 3C2V mimotopes were more effective than p15E in controlling MC38 and B16-F10 tumors. T-cell receptor (TCR) sequencing revealed unique TCR transcripts for mimotopes, but there were no major differences in clonality. These results provide new p15E mimotopes for further vaccine use and illustrate considerations for MHC-I affinity, immunogenicity, and functional efficacy in mimotope design.

**Significance:**

The MHC-I–restricted p15E tumor rejection epitope is expressed in multiple murine cancer lines and is used as a marker of antitumor cellular immunity, but has seen limited success as a vaccine immunogen. An *in vivo* screening approach based on a positional peptide microlibraries is used to identify enhanced p15E mimotopes bearing amino acid mutations that induce significantly improved functional immunogenicity relative to vaccination with the wild-type epitope.

## Introduction

Immunotherapy is a new pillar of cancer treatment and includes a variety of approaches, including cell therapy, immune checkpoint blockade (ICB), and cancer vaccines (1). Cancer vaccines are the oldest of these, are safe and simple to produce, but have achieved relatively limited clinical impact, likely due to the insufficient magnitude of induced functional immune responses (2). Limited T-cell infiltration into solid tumors (3) and immunosuppression in the tumor microenvironment (4) also limit immunotherapy efficacy. Peptide vaccines include the use of short synthetic MHC-I epitopes of just 9–10 amino acids, which represent the minimal determinant required to induce CD8^+^ T-cell responses. For patients with cancer, such short peptides need to be matched in terms of HLA restriction elements, and their immunogenicity can be increased using vaccine adjuvants and peptide delivery systems (5). To further enhance the immunogenicity of short peptides, another approach is to use mimotopes, which here are defined as mutated short peptides that induce a stronger antigen (Ag)-specific CD8^+^ T-cell response against the wild-type epitope than using the wild-type epitope itself as the immunogen.

Several tumor-associated Ags have been targeted using short peptide mimotopes, with superior immunogenicity compared with the wild-type epitope itself. For example, the MART-1_26–35_ with the A27L mutation and gp100_209–217_ with the T210M mutation have been tested in multiple human cancer clinical trials (6, 7). In general, several of the short vaccine mimotopes used in clinical trials have been designed to have a stronger binding affinity with the common HLA-A*0201 restriction element. The binding affinity of short peptides to a wide variety of MHC-Is (including HLA-A*0201) can be predicted using bioinformatics approaches, facilitating such mimotope design. However, MHC-I binding affinity is generally not a strong factor for improving mimotope function, as it does not capture the interaction between the T-cell receptor (TCR) and peptide–MHC complex (pMHC). Because individuals have tens of millions of unique TCRs, there are currently no reliable structural biology methods to systematically predict and improve mimotope design based on the pMHC–TCR interaction. Mimotopes, such as the effective AH1-A5 mimotope against the murine gp70 endogenous viral envelope glycoprotein, have been shown to engage stronger pMHC–TCR interactions, as opposed to higher MHC binding (8). Some approaches to design enhanced mimotope (e-mimotopes) have been put forward based on structural study of pMHC alone (9). Our group recently developed a new approach to develop e-mimotopes based on a nanoparticle vaccine approach, in which peptide microlibraries comprising all approximately 150–180 possible amino acid substitutions of a short MHC-I epitope are used to immunize mice followed directly by tumor challenge (10, 11). This simple screening method is unbiased, does not involve the prediction of MHC-I binding affinity, and can directly identify e-mimotopes that drive improved TCR-pMHC engagement.

The nanoparticle vaccine approach employed in this study involved anchoring short peptides onto the surface of liposomes. This is enabled by appending three histidine residues to the N-terminus of the peptide, followed by incubation with liposomes containing cobalt-porphyrin-phospholipid (CoPoP). The histidine residues interact with cobalt ions in the liposome bilayer, thereby capturing the short peptide Ag (12, 13). In addition, immunostimulatory adjuvants of synthetic monophosphoryl lipid A (PHAD-3D6A) and QS-21 were incorporated and co-delivered with the peptides. Liposomes comprising CoPoP, PHAD-3D6A, and QS-21 (“CPQ”) were previously shown to improve the delivery of short synthetic tumor epitopes to Ag-presenting cells and MHC-I (14) and could lead to strong reversal of large established tumor growth in mice as an immune-based monotherapy with submicrogram peptide dosing (15). This approach was also used to screen peptide microlibraries for short neoepitopes, leading to the identification of the Nesprin-2 L4492R neoepitope in the Renca cancer cell line, which could be used to inhibit tumor growth (16). Adjuvanted CoPoP liposomes were recently tested in humans as a COVID-19 vaccine, resulting in the durable induction of neutralizing antibodies without major safety concerns (17, 18).

In the current study, we developed an e-mimotope for the murine H-2K^b^–restricted p15E tumor epitope (KSPWFTTL; ref. 19). This epitope, located at residues 604–611 of the p15E protein, was identified as an immunodominant tumor rejection epitope of endogenous ecotropic murine leukemia virus (MuLV; ref. 20). The p15E is expressed by MC38 and B16-F10 cancer cells, which are frequently used for syngeneic murine tumor models (21). Although commonly used as a marker for Ag-specific, antitumor CD8^+^ T cell–mediated immunity (22–25), p15E has rarely been reported as a vaccine epitope capable of inhibiting tumor growth. Previous studies have shown that p15E immunization did not lead to tumor rejection in a prophylactic setting using a peptide viral capsid conjugate approach (26, 27), whereas the use of a recombinant vaccinia viral vector resulted in only a partial reduction of lung metastases (20). More recently, TCR sequencing of B16-F10 tumor-infiltrating lymphocytes revealed p15E-specific transcripts which were then used to together with a yeast display system to generate mimotopes that induced modest tumor delay with immunization (28). As described here, we found that the wild-type p15E epitope, despite being delivered with our potent peptide nanoparticle vaccine system, was virtually non-immunogenic and non-functional. However, e-mimotopes could be identified that conferred improved immunogenicity and antitumor activity.

## Materials and Methods

### Liposome Preparation

Lipids for liposome production included 1,2-dipalmitoyl-sn-glycero-3-phosphatidylcholine (DOPC, Corden catalog no. LP-R4-076), cholesterol (PhytoChol, abbreviated Chol herein, Wilshire Technologies/Evonik), and synthetic monophosphoryl hexa-acyl lipid A, 3-deacyl (PHAD 3D6A, abbreviated PHAD herein, Avanti, catalog no. 699855P). CoPoP and PoP were synthesized as described previously (14). QS-21 was obtained from Desert King. CoPoP/PHAD/QS21 (abbreviated as CPQ) liposomes were synthesized by the ethanol injection method followed by nitrogen-pressurized lipid extrusion, as published previously (29). Lipids were weighed at the mass ratio of DOPC: Chol: CoPoP: PHAD (20:5:1:0.4), or DOPC: Chol: PoP: PHAD (20:5:1:0.4) and dissolved in 1 mL 60°C prewarmed ethanol before sonication and dilution with 4 mL of prewarmed PBS at 60°C. Liposomes were passed through a 0.45 µm filter and then extruded through stacks of 200, 100, and 80 nm membrane filters at 55°C using a nitrogen-pressurized liposome extruder (Northern lipids). The extruded liposomes were dialyzed in PBS at 4°C overnight. QS-21 was then incubated with CoPoP-formulated liposomes at a CoPoP: QS-21 mass ratio of 1:0.4 overnight at 4°C.

### Peptide and Vaccine Preparation

His-tagged peptides three N-terminal histidine residues, included E7 (HHH-RAHYNIVTF), wild-type p15E (HHH-KSPWFTTL), p15E-3M (HHH-KSMWFTTL), p15E-3C (HHH-KSCWFTTL), p15E-3C2V (HHH-KVCWFTTL) and others were synthesized by GenScript at greater than 95% purity. Peptide microlibraries were synthesized by Genscript at the crude purity level as mixtures of 20 peptides randomized at a single residue of p15E. Peptides were reconstituted using sterile water or PBS to 1 mg/mL peptide stock solution. To prepare vaccines, 2 µg peptide and 8 µg of CoPoP-containing CPQ liposomes were incubated at room temperature for at least 1 hour before dilution with PBS to a final concentration of 2 µg total peptide per 50 µL vaccine per mouse per injection. Each injection also included 8 µg CoPoP, 3.2 µg QS-21, and 3.2 µg PHAD. To prepare the vaccine for peptide position library screening, 5 µg of positional library peptides were incubated with CPQ liposomes (CoPoP: peptides at a weight ratio of 4: 1) for each mouse per injection. Liposome size and polydispersity index (PDI) before and after incubation of peptides were measured by adding 2 µL of samples to 1 mL PBS and using dynamic light scattering.

### Peptide Loading Assessment

Peptides were coincubated with CoPoP or PoP liposomes with a mass ratio of CoPoP: peptide at 4:1. After 1-hour incubation at room temperature, samples were centrifuged at 26,000 × *g* at 4°C overnight. Supernatants containing unbound peptide were collected, and peptide concentration was determined using a micro bicinchoninic acid (BCA) Protein Assay Kit (Thermo Fisher Scientific, catalog no. 23235) at an absorbance of 562 nm. The percentage of peptide loaded = [1 − (testing sample peptide absorbance – liposome background absorbance)/(free peptide absorbance – PBS background absorbance)] × 100.

### MHC-I Binding

RMA-S cells were provided by Dr. Scott I. Abrams from Roswell Park Comprehensive Cancer Center. RMA-S cells were cultured in RPMI medium supplemented with 10% FBS and 1% 100 U/mL penicillin. Next, 100 µL of 2 × 10^6^ RMAs cells were suspended in RPMI-only medium and incubated with 100 µL of 4 µg peptide-bound liposomes. After incubation at 26 °C with 5% CO_2_ for 2 hours, cells were washed with 200 µL sterile PBS and centrifuged at 500 × *g* for 5 minutes, twice. Cells were incubated in 200 µL RPMI medium at 37°C with 5% CO_2_ and were collected at different timepoints and washed with sterile PBS. Collected cells were centrifuged at 500 × *g* for 5 minutes and resuspended in 200 µL 4% formaldehyde (Alfa Aesar, catalog no. J60401-AK) at room temperature for 20 minutes. Fixed cells were washed once with sterile PBS, centrifuged at 500 × *g* for 5 minutes, and resuspended in 200 µL of PBS. After all cells were collected and fixed at different timepoints, cells were centrifuged at 500 × *g* for 5 minutes to remove supernatant. Cells were then incubated with 60 µL PE-labeled anti-mouse H-2K^b^ (BD Pharmingen, catalog no. 553570) at 200 times final dilution in FACS buffer (PBS containing 0.5% BSA) for 30 minutes with constant shaking. The supernatant was discarded, and the cells were resuspended in 200 µL FACS buffer for flow cytometry. The percentage of signal loss = (1 – PE signal intensity at different timepoints/initial PE signal intensity) × 100.

### Murine Studies

C57BL/6 mice (6–7 weeks old, female) were purchased from Jackson Laboratory and maintained at the Comparative Medicine and Laboratory Animal Facilities. Approval was granted by the Institutional Animal Care and Use Committee (IACUC) of the University at Buffalo, State University of New York (IACUC Protocol # BME13028Y). All mice were acclimated for 7 days before the experiments.

Mice received indicated doses of the vaccine as described in figure captions via intramuscular immunization at 7-day interval. Blood samples were collected from mice cheek by lancet on day 14. For tumor challenge studies, MC38 cells (Kerafast catalog no. ENH204-FP) and B16-F10 (American Type Culture Collection catalog no. CRL-6475) cells were cultured in DMEM culture medium supplemented with 10% FBS and 1% penicillin/streptomycin at 37°C in 5% CO_2_ incubator. TC-1 cells (kindly provided by Dr. Jorge Gomez-Gutierrez, University of Louisville, Kentucky, USA) were cultured in RPMI1640 culture medium supplemented with 10% FBS and 1% penicillin/streptomycin at 37°C in 5% CO_2_ incubator. On the day of tumor inoculation, cells were collected, counted, and resuspended in cold RPMI medium. Mice were anesthetized by inhalation of isoflurane and were inoculated with 1 × 10^6^ MC38, 1 × 10^5^ B16-F10 cells, or 1 × 10^5^ TC-1 cells for prophylactic tumor challenges and 1 × 10^5^ MC38 for therapeutic tumor challenge at the left lower flank subcutaneously and monitored accordingly. Mice were monitored and euthanized when the tumor length reached 15 mm or showed signs of sustained ulceration.

### CTL Assay

Mice were immunized with two intramuscular injections of 2 µg peptide in CPQ formulated vaccines a 7-day interval. Splenocytes were harvested 7 days after the second immunization and separated with 70 µm cell strainers before treatment with red blood cell (RBC) lysis buffer (Invitrogen). Mouse IL2 Recombinant Protein (PeproTech, catalog no. 212-12; 10 IU/mL) and 10 µg/mL Ags were added to splenocytes and cultured for 5 days with DMEM culture medium supplemented with 10% FBS and 1% penicillin/streptomycin at 37°C in 5% CO_2_ incubator. Counted splenocytes (effector cells) were seeded into sterile 96-well plates at the indicated effector:tumor cell (E:T) ratio. A fixed number of 5,000 MC38 tumor cells (target cells) were stimulated with 10 µg/mL p15E wild-type peptide for 1 hour at 37°C in 5% CO_2_ incubator. Target cells and effector cells were coincubated at E:T ratios 50:1, 25:1, 12.5:1, and 6.25:1 for 4 hours at 37°C in 5% incubator. The 96-well plate was centrifuged for 5 minutes at 1,350 rpm. A total of 50 µL of supernatant was collected, and the amount of lactate dehydrogenase (LDH) released in each well was calculated using the CytoTox 96 Non-Radioactive Cytotoxicity Assay (Promega). The percentage of cell killing = (experimental sample release–effector cell spontaneous release–target cell spontaneous release)/(target cell maximum release − target cell spontaneous release) × 100.

### IFNγ ELISPOT Assay

The precoated Mouse IFNγ Single-Color ELISPOT kit (ImmunoSpot) was used to quantify IFNγ production. On day 7 after second vaccination, mouse splenocytes were harvested and separated with 70 µm cell strainers before being treated with RBC lysis buffer. After wash with PBS and resuspend in 1 mL PBS, 3 × 10^5^ splenocytes in 100 µL CTL medium were incubated with 100 µL 20 µg/mL indicated stimulating peptide for 24 hours at 37°C, 5% CO_2_ incubator. All remaining steps strictly followed the assay kit protocol.

### CD8^+^ and CD4^+^ T-cell Depletion

C57BL/6 mice received intraperitoneal injection of 400 µg InVivoMAb anti-mouse CD8α antibody (Bio X Cell, catalog no. BE0004-1) or 400 µg InVivoMAb anti-mouse CD4 antibody (Bio X Cell, catalog no. BE0003-1) per mouse per injection 2 days and 1 day before tumor inoculation. On day 4 and day 8 after tumor inoculation, mice received 200 µg InVivoMAb anti-mouse CD8α or 200 µg InVivoMAb anti-mouse CD4 per mouse per intraperitoneal injection. All antibodies were diluted to 100 µL volume with PBS for per mouse per injection.

### Flow Cytometry

A total of 60 µL blood was collected and stained with 10 µL E7-specific or p15E-specific tetramer (both provided by NIH tetramer core facility) to a final 500 times dilution for 1 hour on ice with constant shaking. Aqua fluorescent reactive dye for live and dead staining (Invitrogen, catalog no. L34957) in a final 500 times dilution, FITC anti-mouse CD4 Antibody (BioLegend, catalog no. 100406) and APC anti-mouse CD8a Antibody (BioLegend, catalog no. 100712) in a final 200 times dilution was added to the testing samples to a total volume of 100 µL and incubated for 30 minutes on ice with constant shaking. After one wash with cold PBS and centrifugation at 1,350 rpm for 3 minutes, the cells were resuspended in 200 µL FACS buffer and ready for examination by using BD LSRFortessa X-20 cytometer. Data were processed by Flowjo (version 10).

### Peptides MHC Class I Binding Prediction

Peptide binding affinity to MHC-I proteins were predicted by using following servers: the NetMHC 4.0 server (https://services.healthtech.dtu.dk/services/NetMHC-4.0/), the NetMHCpan 4.0 server (https://services.healthtech.dtu.dk/services/NetMHCpan-4.0/), and the NetMHCpan 4.1 server (https://services.healthtech.dtu.dk/services/NetMHCpan-4.1/).

### TCR Sequencing

The spleens of immunized mice were harvested, separated, and treated with RBC lysis buffer. Whole splenocytes were washed and reconstituted in 1 mL of PBS buffer. The p15E-specific tetramer was diluted in 0.75 mL FACS buffer and added to 1 mL splenocytes to a final 500 times tetramer dilution. After 1 hour of incubation on ice with constant shaking, 200 times final diluted APC-CD8 T-cell antibodies and 500 times final diluted aqua fluorescent reactive dye in 750 µL FACS buffer were added and incubated on ice with constant shaking for 30 minutes. Splenocytes were washed with PBS, reconstituted with 2 mL of FACS buffer, and ready for cell sorting by using SONY MA900 Cell Sorter. Sorted tetramer-specific CD8^+^ T cells were collected in 200 µL 0.025 mol/L HEPES buffer supplemented with 2% FBS. DNA extraction was performed using the DNeasy Blood & Tissue Kit (QIAGEN) and concentrated using a Vacuum Concentrator. DNA concentration was determined on the basis of the absorbance of A260/A280 and A260/A230 by using Nanodrop one C (Thermo Fisher Scientific). Amplification and sequencing of TCRβ CDR3 regions were performed using the ImmunoSEQ immune profiling system at the survey level (Adaptive Biotechnologies). T cell repertoires, comprising all detected CDR3 sequences with annotated V and J gene segment identifications, were downloaded directly to the ImmunoSEQ Analyzer from Adaptive Biotechnologies. The metrics of the complete TCR repertoire in each sample, including the number of productive rearrangements, productive clonality, and clonal frequencies, were determined using the ImmunoSEQ Analyzer software and confirmed using the LymphoSeq package [Coffey, D., Lymphoseq: Analyze High-Throughput Sequencing of T and B Cell Receptors. *R package version 1.14.0.* 2019.]. The repertoires were analyzed using the LymphoSeq package and custom scripts in the R statistical software environment. Differential clonotype abundance between groups was determined using DESeq2 (30).

### Data Availability and Statistical Analysis

The data generated in this study are available within the article and its Supplementary Data files. Data were analyzed using GraphPad Prism 9, as indicated in the figure captions. Values are reported as mean ± SD.

## Results

### The p15E Epitope is Ineffective as a Synthetic Vaccine Immunogen

**FIGURE 1:**
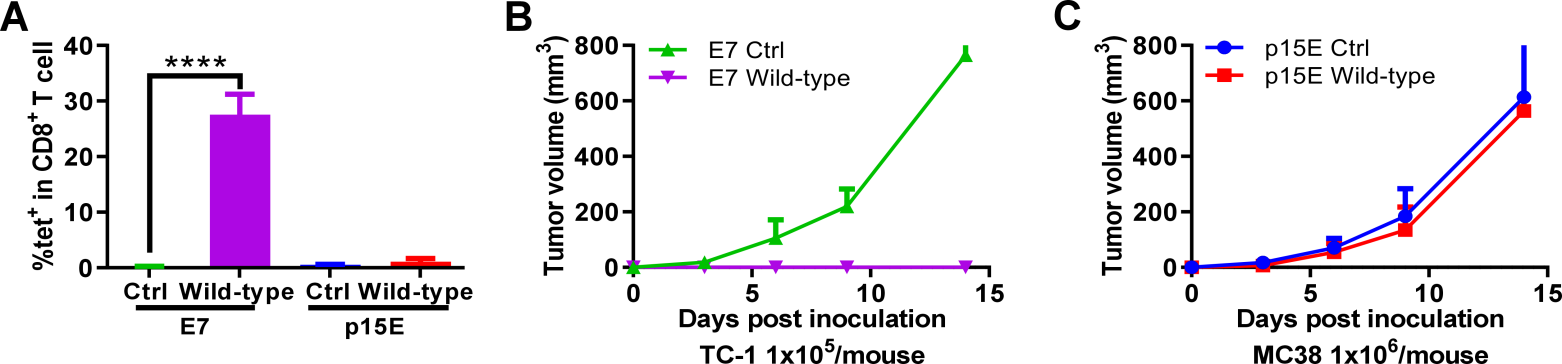
The wild-type p15E short peptide is poorly immunogenic and does not inhibit tumor growth. C57BL/6 mice were vaccinated intramuscularly with 2 μg of E7 or p15E peptide displayed on CPQ liposomes (along with 8 μg CoPoP, 3.2 μg PHAD and 3.2 μg QS-21) on day 0 and 7; then blood was collected for Ag-specific CD8^+^ T-cell tetramer staining on day 14 and E7 vaccinated mice were challenged with 1 × 10^5^ TC-1 cells and p15E vaccinated mice were challenged with 1 × 10^6^ MC38 cells. (**A**) Ag-specific CD8^+^ T-cell percentages in CD8^+^ T cells in blood. TC-1 (**B**) and MC38 (**C**) tumor volume in non-vaccinated control (Ctrl) mice, or mice immunized with the E7 or p15E (wild-type) peptides. Statistical analysis shows unpaired *t* test. ****, *P* < 0.0001. Error bars show mean + SD for 3 mice per group.

Initially, we compared immunization using the short p15E peptide (KSPWFTTL) with the short E7_49–57_ (RAHYNIVTF) peptide. Derived from the E7 oncoprotein of the human papillomavirus type 16 (HPV-16), the E7 protein is expressed in the TC-1 murine tumor cell line. We previously showed that the short E7 epitope can effectively inhibit and reverse established tumor growth following immunization with the CPQ liposome adjuvant system (15). Both p15E and E7 peptides were modified with three histidine residues on the N-terminus for display on the CPQ liposomes. As shown in Fig. 1A, following intramuscular priming and boosting with the E7/CPQ vaccine, approximately 28% of CD8^+^ T cells in the peripheral blood became E7-specific, based on tetramer staining. In contrast, p15E/CPQ immunization did not result in any measurable increase in Ag-specific CD8^+^ T-cell population. The E7-immunized mice completely rejected the subsequent tumor challenge with TC-1 tumor cells (Fig. 1B). Conversely, p15E-immunized mice did not reject the MC38 tumor challenge and did not exhibit delayed tumor growth compared with unimmunized mice (Fig. 1C). Therefore, the p15E peptide epitope showed no evidence of immunogenicity and was inefficient as a tumor vaccine epitope under these conditions despite the use of a potent adjuvanted vaccine nanoparticle approach.

### Identification of p15E Mimotopes via a Two-step Peptide Library Screening Approach

**FIGURE 2:**
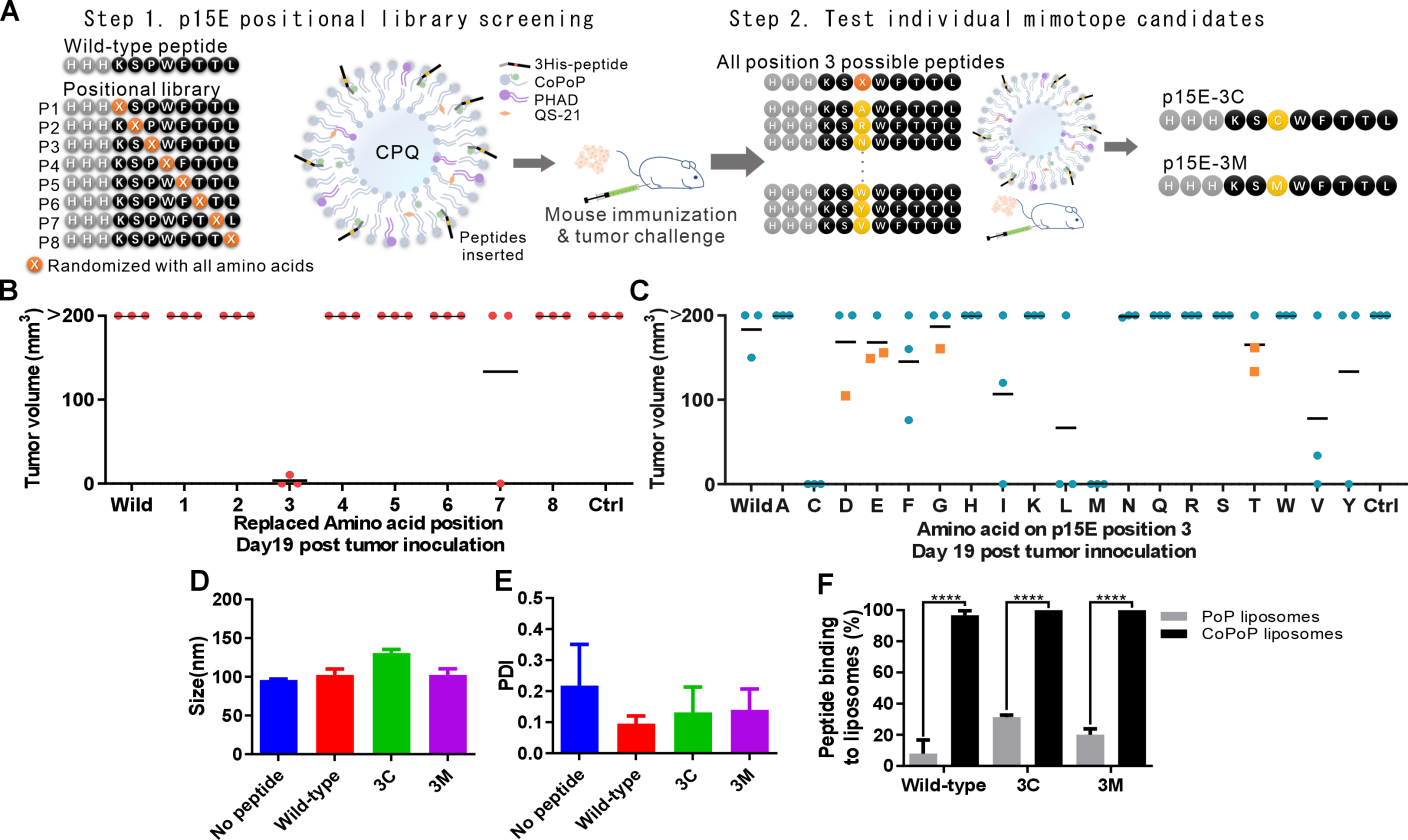
Functional screening of positional peptide microlibraries reveals 3M and 3C substitutions as p15E e-mimotopes. **A,** Schematic shows the strategy of two-step e-mimotope screening. Mice were vaccinated with peptide position library vaccines (**B**) or individual peptide vaccines (**C**) with the position 3 amino acid being replaced, as indicated. Vaccinations occurred on days 0 and day 7, followed by inoculation of 1 × 10^6^ MC38 cells on day 14. Tumor volume on day 19 after tumor inoculation was recorded. The square symbol in C indicates the mouse was euthanized because of tumor ulceration before day 19. Size (**D**) and PDI (**E**) of CPQ liposomes with the indicated peptide. **F,** Binding of peptides to PoP (lacking cobalt) or CoPoP liposomes after incubation at room temperature for 1 hour. Statistical analysis applied using the unpaired *t* test. ****, *P* < 0.0001. Error bars show mean + SD for three independent experiments.

To determine whether enhanced p15E mimotopes could be identified, eight p15E position libraries were screened using a CPQ liposome platform. For each position of the p15E peptide, the wild-type amino acid was replaced by a random library of other 19 common amino acids (Fig. 2A, left). Peptides were synthesized with three histidine residues on the N-terminus of the peptide sequence for display on CPQ liposomes. Positional peptide libraries were prepared as nanoparticle vaccines by incubating them with CPQ liposomes at room temperature for 1 hour before diluting with PBS. After two immunizations on days 0 and 7 separately, mice were inoculated with 1 × 10^6^ MC38 cells on day 14. The tumor volumes of the mice 19 days later are shown in Fig. 2B. All mice immunized with the wild-type p15E peptide had tumor volumes >200 mm^3^, as did non-immunized control mice. Strikingly, mice that had been immunized with the p15E peptide library randomized at position 3 showed tumor growth inhibition, with 2 of the 3 mice in this group being free of detectable tumor growth and the remaining mouse bearing a tumor volume <20 mm^3^. Mice immunized with nanoparticle peptide libraries that had been randomized at other residues of p15E, besides position 3, did not reject tumor growth.

The second step of the mimotope screen sought to assess which individual amino acid substitutions within the position 3 (P3) library were responsible for the e-mimotope function. CPQ liposomes were incubated with 19 mimotopes separately, with only one amino acid at position 3 being replaced (Fig. 2A, right). Mice received these single mutation mimotope candidate vaccines on days 0 and 7, and were challenged with 1 × 10^6^ MC38 cells on day 14. Nineteen days after tumor inoculation, mice that received the vaccine containing the peptide P3 amino acid proline replaced by cysteine or methionine showed tumor growth inhibition (Fig. 2C). All other groups, including the p15E wild-type peptide group, induced weaker tumor growth–inhibiting effects, as evidenced by mice showing tumor growth. Thus, replacing proline at P3 of the p15E wild-type peptide with cysteine or methionine enhanced the antitumor response with vaccination.

When CPQ liposomes displayed the p15E wild-type peptide, the p15E-3C (3C) mimotope, or the p15E-3M (3M) mimotope, only the 3C mimotope slightly increased the liposome size from approximately 100 nm to approximately 130 nm (Fig. 2D). All Ag-loaded liposomes were generally homogeneous with an average PDI below 0.3 (Fig. 2E). Next, the binding of p15E peptides to liposomes was confirmed (Fig. 2F). Liposomes that included cobalt-free PoP did not effectively sequester the short peptides, with only 10%–30% peptide binding observed. In contrast, when CoPoP was included in the liposomes, over 90% of the 3C and 3M mimotopes bound the liposomes. As expected, CoPoP was required for effective liposome display of the short peptides. Overall, the CPQ liposomes effectively captured these short peptides and formed homogeneous particles.

### Mimotope MHC-I Binding and Function

**FIGURE 3:**
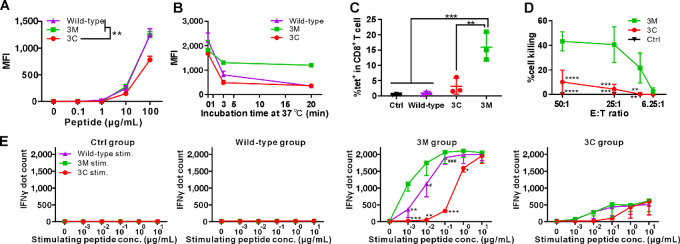
The p15E-3M mimotope induces improved cytotoxic T-cell responses *in vitro*. **A,** Mean fluorescence intensity (MFI) of the indicated peptides bound to RMA-S cells after staining with PE-labeled anti-mouse H-2K^b^ antibody. **B,** Binding stability of the indicated peptide with RMA-S MHC-I molecules. Mice were immunized with 2 µg of the indicated peptide liposomal vaccine per injection on days 0 and day 7. **C**, The percentage of p15E-specific CD8^+^ T cells in all CD8^+^ T cells from blood tested on day 14. Splenocytes were collected on day 14 for cytotoxic CD8^+^ T-cell generation and lytic activity evaluation from the indicated groups (**D**). Splenocytes were stimulated with the different peptides as indicated. **E**, IFNγ ELISPOT assays were recorded. Datapoints represent mean values of 3 mice from the same group for A, B, and E; represent mean values of four mice for D. Datapoints represent individual values for each mouse for C. Statistical analysis applied one-way ANOVA followed by Tukey multiple comparisons tests. *, **, ***, and **** indicating *P* < 0.05, 0.01, 0.001, and 0.0001, or respectively compared with the 3M group in E; ^#^,^###^ indicating *P* < 0.05, 0.001, respectively compared with 3C group in E. Error bars show mean +, ±, or − SD.

To investigate the mechanisms underlying the enhanced tumor growth–inhibiting effects, peptide and MHC-I binding affinity, Ag-specific CD8^+^ T-cell frequency, cytotoxic activity, and IFNγ production were assessed. The p15E wild-type peptide is MHC-I H-2K^b^ restricted. By incubating the p15E wild-type peptide and the 3C or 3M mimotopes separately with the TAP-deficient RMA-S cell line, a PE-labeled anti-mouse H-2K^b^ antibody was used to capture the MHC-I H-2K^b^ molecule that presented the bound epitopes, stabilizing the surface expression of MHC-I. All three tested MHC-I epitopes showed increased presentation on H-2K^b^ from 1 to 100 µg/mL of peptide, although the 3C mimotope showed a lower binding affinity than the wild-type epitope and the 3M mimotope, with a significant difference observed at 100 µg/mL (Fig. 3A). As discussed below, this is consistent with 3C having diminished MHC-I binding affinity. The peptide-MHC binding stability was then tested by incubating these peptides with RMA-S cells at 37°C, which is the temperature that the H-2K^b^ molecule dissociates from RMA-S cells (31). At different incubation times, RMA-S cells were collected for flow cytometry analysis of PE signal intensity. The PE signal intensity of all peptide-treated RMA-S cells decreased with incubation time. Compared with the initial PE signal intensity of each group, approximately 70% of the PE signal decreased from the 3C group, 30% from the 3M group, and approximately 50% from the wild-type group in the first 3 minutes (Fig. 3B). With a longer incubation period of 20 minutes at 37°C, the 3M group still retained approximately 70% of the initial H-2K^b^ reactivity, whereas the 3C group retained only approximately 30%, and the wild-type group fell to approximately 20%.

Blood was collected after two immunizations and stained with an anti-CD8 antibody and a tetramer specific for the wild-type p15E peptide. The wild-type showed no significant difference compared with the untreated control group in the percentage of tetramer-specific CD8^+^ T cells among all CD8^+^ T-cell populations. 3C induced a slightly higher level, but it was not significantly more p15E-specific CD8^+^ T cells compared with control. In contrast, the 3M group had a significant increase compared with the wild-type peptide-specific CD8^+^ T-cell population to approximately 15% on average compared with the control (0.5%), wild-type (1%), and 3C (2.6%) groups (Fig. 3C). Representative gating strategy is shown in Supplementary Fig. S1A. Splenocytes were recovered after two immunizations and cultured with the corresponding peptide plus IL2 for 5 days to generate CD8^+^ T cells for lytic assays. These effector cells were then coincubated with peptide-bearing tumor target cells at the indicated E:T ratio. Cell death was quantified using the LDH release assay. Effector cells from the 3M group lysed >40% of the target cells at E:T ratios of 50:1 and 25:1, whereas effector cells from the 3C group lysed approximately 10% and 5% of target cells at the same ratios (Fig. 3D). IL2 stimulated splenocytes from the untreated control group did not show detectable target cell lysis.

Next, we measured IFNγ production to assess immunogenicity using an ELISPOT assay. Splenocytes of immunized mice were restimulated with the indicated peptide at concentrations ranging from 0 to 10 µg/mL, followed by the quantification of IFNγ levels. As shown in Fig. 3E, the control and wild-type groups did not produce detectable IFNγ after restimulation with the wild-type, 3M, or 3C peptides. In contrast, both the 3M and the 3C groups showed increased IFNγ production in a dose-dependent manner. In the 3M vaccinated group, the 3M peptide induced the highest level of IFNγ, with a significant difference between the wild-type and 3C groups, especially at low restimulation peptide concentrations (Fig. 3E, middle right). In the 3C after immune splenocytes, restimulation with the 3M or wild-type peptide led to a higher IFNγ production compared with restimulation with the 3C peptide, although there was no statistically significant difference between any of the peptides (Fig. 3E, right).

Overall, the 3M mimotope showed high binding affinity with the MHC-I molecule as well as enhanced binding stability compared with the wild-type peptide. The 3M mimotope also induced a higher frequency of wild-type peptide Ag-specific CD8^+^ T cells, stronger cytotoxic activity, and led to a higher production of IFNγ.

### p15E-3C Mimotope has Enhanced Antitumor Function in a Therapeutic Vaccine Setting

**FIGURE 4:**
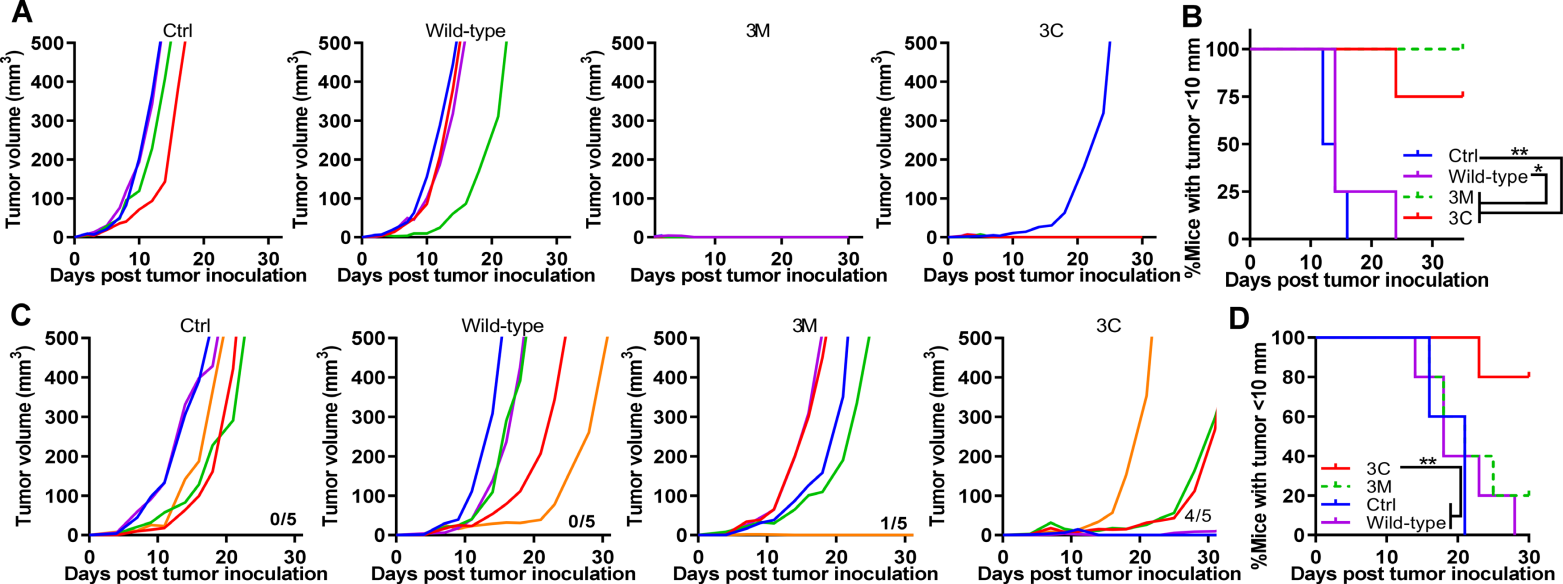
Efficacy of p15E-3C and p15E-3M e-mimotope vaccines at submicrogram peptide doses in prophylactic and therapeutic MC38 tumor challenges. Mice were immunized intramuscularly with CPQ liposomes containing 0.8 µg PHAD, 0.8 µg QS-21, and 0.5 µg p15E wild-type, p15-3C, or p15-3M on days 0 and 7 and were challenged with 1 × 10^6^ MC-38 cells subcutaneously on day 14 (*n* = 5 mice per group). Individual tumor volumes (**A**) and the percentage of mice with tumor length under 10 mm (**B**). For therapeutic challenge, mice were inoculated with 1 × 10^5^ MC-38 cells on day 0 and immunized with 0.5 µg p15E wild-type or 3C or 3M liposomal vaccine on day 2 and 9 (*n* = 5 mice per group). Individual tumor volumes (**C**) and the percentage of mice with a tumor <10 mm in diameter (**D**). Statistical analysis by log-rank (Mantel–Cox) test. * and ** indicates *P* < 0.05 and 0.01, respectively.

To assess the tumor growth–inhibiting effects of these e-mimotope candidates *in vivo*, wild-type p15E peptide, p15E-3C, and p15E-3M were separately loaded onto CPQ liposomes. After two immunizations separated by 7-day intervals, mice were inoculated with 1 × 10^6^ MC38 cells 7 days after the second immunization. The unvaccinated control group and wild-type peptide-vaccinated groups showed tumor growth in all mice (Fig. 4A). All 5 mice in the 3M-treated group were tumor-free, while one of the 5 mice from the 3C-treated group grew a tumor. By days 17 and 23 after tumor inoculation, all mice in the untreated control and wild-type groups had tumor diameters of >10 mm (Fig. 4B). In contrast, only one mouse in the 3C group had a tumor diameter >10 mm by day 23, whereas the remainder were tumor-free. log-rank tests showed significant differences among the 3M and 3C with control and wild-type groups. No statistically significant difference was observed between the 3M and 3C groups.

In the therapeutic setting, mice were inoculated with 1 × 10^5^ MC38 cells on day 0 and immunized on days 2 and 9 with the indicated vaccines. The numbers above the *x*-axis in Fig. 4C indicate the number of mice that survived until day 30. Among the five mice tested from each group, all the control and wild-type groups showed tumor growth, while one mouse from the 3M group was tumor-free. In the 3C group, 1 mouse was tumor-free, 1 had a small tumor of approximately 10 mm^3^, and 2 showed slowly growing tumors. On the basis of the percentages of mice with tumor diameters <10 mm, the 3C group showed significantly higher tumor growth-inhibiting activity than the control and wild-type groups, but not in the 3M group (Fig. 4D). Overall, the 3M and 3C mimotope vaccine groups showed strong antitumor effects in the prophylactic MC38 tumor challenge setting, whereas the less immunogenic 3C mimotope best inhibited tumor growth and prolonged survival in the therapeutic MC38 setting.

**FIGURE 5:**
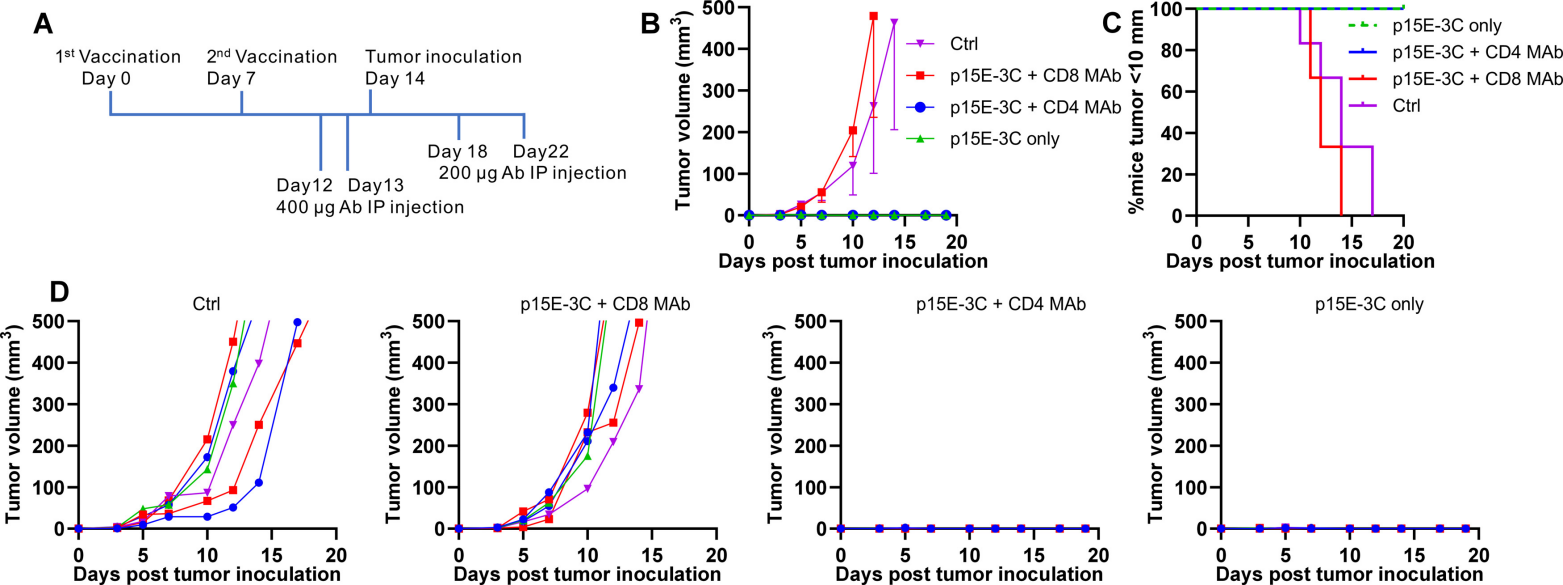
p15E-3C mimotope-induced inhibition of MC38 tumor growth is CD8^+^ T-cell dependent. Mice received 2 µg p15E-3C liposomal vaccine on days 0 and 7 before challenge with 1 × 10^6^ MC-38 cells on day 14 (*n* = 6). **A,** Schedule of injections and tumor inoculation. Mice received 400 µg CD8 MAb or CD4 MAb on day 12 and day 13 as indicated; 200 µg CD8 mAb or CD4 mAb on day 18 and day 22 as indicated. **B,** Average tumor volume of indicated groups. **C,** Percentage of mice with tumor length shorter than 10 mm. **D,** Tumor volumes of individual mice from indicated groups. Datapoints represent mean values of 6 mice from the same group for B. Error bars show mean − SD.

To confirm whether antitumor activity of the p15E-3C e-mimotope was CD8^+^ T cell–dependent as expected (because the short e-mimotope is expected to bind only MHC-I), a T-cell depletion study was carried out. Anti-CD4 T-cell antibodies (CD4 mAb) and anti-CD8 T-cell antibodies (CD8 mAb) were used to deplete CD4 and CD8 separately. Flow cytometry confirmed effective depletion of CD4 or CD8 T cells on day 14 (Supplementary Fig. S2). As shown in Fig. 5A, besides the untreated control group, all mice received two injections of p15E-3C mimotope liposomal vaccines (p15E-3C only group). Four injections of anti-CD4 antibodies (p15E-3C + CD4 MAb group) or anti-CD8 antibodies (p15E-3C + CD8 mAb group) were applied to deplete CD4^+^ or CD8^+^ T cells as indicated. Mice from the control group and p15E-3C + CD8 MAb group showed increased MC38 tumor growth, while mice from the p15E-3C only group and p15E-3C + CD4 MAb group remain tumor free till the day 20 (Fig. 5B). No mice from the p15E-3C only group and p15E-3C + CD4 MAb group showed tumor length over 10 mm (Fig. 5C). Tumor volume of individual mouse was shown in Fig. 5D. In summary, after two vaccinations, p15E-3C mimotope vaccinated mice dependent on CD8^+^ T cells instead of CD4^+^ T cells to inhibit MC38 tumor growth.

### Identification of Additional Short p15E Mimotopes that Incorporate the 3C Mutation

**FIGURE 6:**
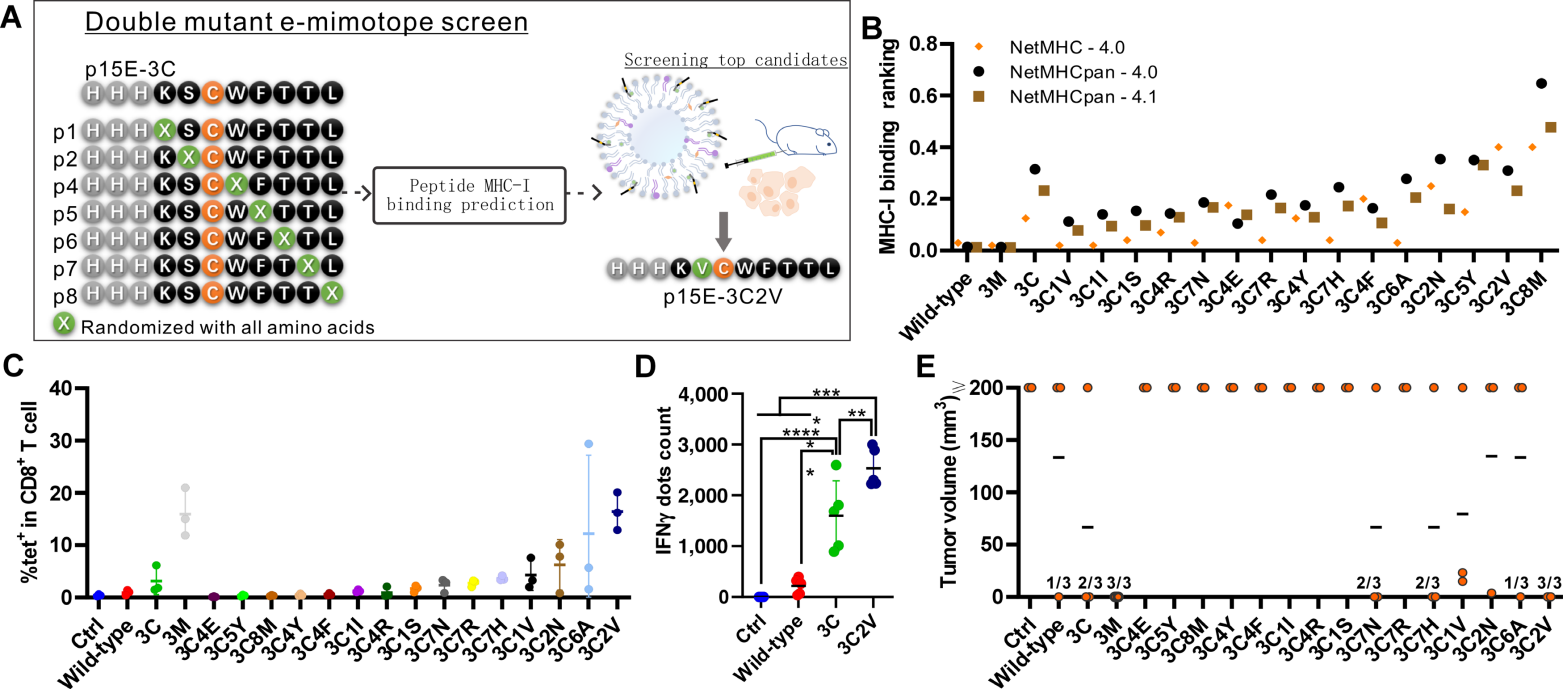
Generation of two-mutation p15E mimotopes that incorporate the 3C mutation. **A,** Strategy of e-mimotope screening for p15E-3C. (**B**) Peptide and MHC-I binding prediction by NetMHC algorithms. After vaccination on days 0 and day 7 with the indicated peptides with CPQ, blood was collected on day 14 for wild-type p15E-specific CD8^+^ T-cell frequency (**C**) as measured by tetramer staining. On day 14, 1 × 10^6^ MC38 cells were inoculated subcutaneously. Mice received 2 µg peptide of the indicated liposomal vaccine on days 0 and day 7, splenocytes were harvested on day 14 and stimulated with the p15E wild-type peptide for IFNγ production by ELISOPOT (*n* = 5; **D**). On day 19 after tumor inoculation, tumor volume (**E**) was recorded (*n* = 3). Datapoints represent individual values of 3 mice from the same group in C and E or 5 mice from the same group in D. Statistical analysis applied one-way ANOVA followed by Tukey multiple comparisons tests. *, **, *** and **** indicating *P* < 0.05, 0.01, 0.001, and 0.0001, respectively. Error bars show mean ± SD.

The 3C mimotope vaccine induced a lower frequency of Ag-specific CD8^+^ T-cell responses (Fig. 3C) but a strong magnitude of antitumor activity (Fig. 4D) compared with the 3M mimotope. Thus, based on the 3C mutation, we assessed further mimotope candidates bearing additional mutations to potentially further improve immunogenicity and antitumor activity. To that end, we first used peptide-MHC binding prediction methods to rank binding affinity of mimotope candidates (Fig. 6A). According to the predicted MHC-I binding affinity ranking, the top 15 new mimotopes based on the 3C (KSCWFTTL) mimotope were selected from the 133 candidates (Fig. 6B). They are all predicted to be strong binders to MHC-I molecule and were named according to the amino acid replaced and its position within the sequence. For example, the 3C2V (KVCWFTTL) mimotope indicates the third amino acid is cysteine and the second amino acid is valine.

Following immunization with the mimotopes, the frequency of Ag-specific CD8^+^ T cells was determined with wild-type p15E tetramer reactivity was determined by flow cytometry. In the 3C2V mimotope-immunized group, the frequency of CD8^+^ T cells reactive against the wild-type peptide was significantly increased relative to the control group, wild-type group, and the 3C group, but comparable to the 3M group (Fig. 6C). The 3C2V mimotope-immunized mice also showed significantly increased IFNγ production compared with the wild-type and 3C peptide groups (Fig. 6D). All 3 mice from the 3C2V group or the 3M group inhibited MC38 tumor growth in a prophylactic setting, while 1 mouse from both the wild-type and 3C6A groups, and 2 from the 3C, 3C7N, and 3C7H groups were tumor-free (Fig. 6E). While this study was not powered to determine whether any differences were present between mimotopes, the 3C2V mimotope showed enhanced p15E-reactive immunogenicity relative to 3C, and the potential for inducing long-lived memory responses. Mice that survived the initial tumor challenge (Fig. 6E) and 5 new control mice were (re)challenged 100 days after the initial tumor inoculation with 1 × 10^6^ MC38 (Supplementary Fig. S3A). All 3 mice from the 3C2V group (Supplementary Fig. S3E), the only one mouse from the wild-type (Supplementary Fig. S3B), and the only one mouse from the 3C6A group (Fig. 3F) were tumor-free and remained tumor-free through day 60 of the observational period. None of the other groups showed complete inhibition of tumor growth in this rechallenge setting (Supplementary Fig. S3C–S3D and S3G–S3H). The 3M and 3C mimotope-immunized mice showed better survival than the wild-type group during the first tumor challenge but did not provide full protection during the tumor re-challenge. Overall, the 3C2V mimotope provided long-term protection against MC38 tumor challenges.

### p15E Mimotope Efficacy in Multiple Tumor Models

**FIGURE 7:**
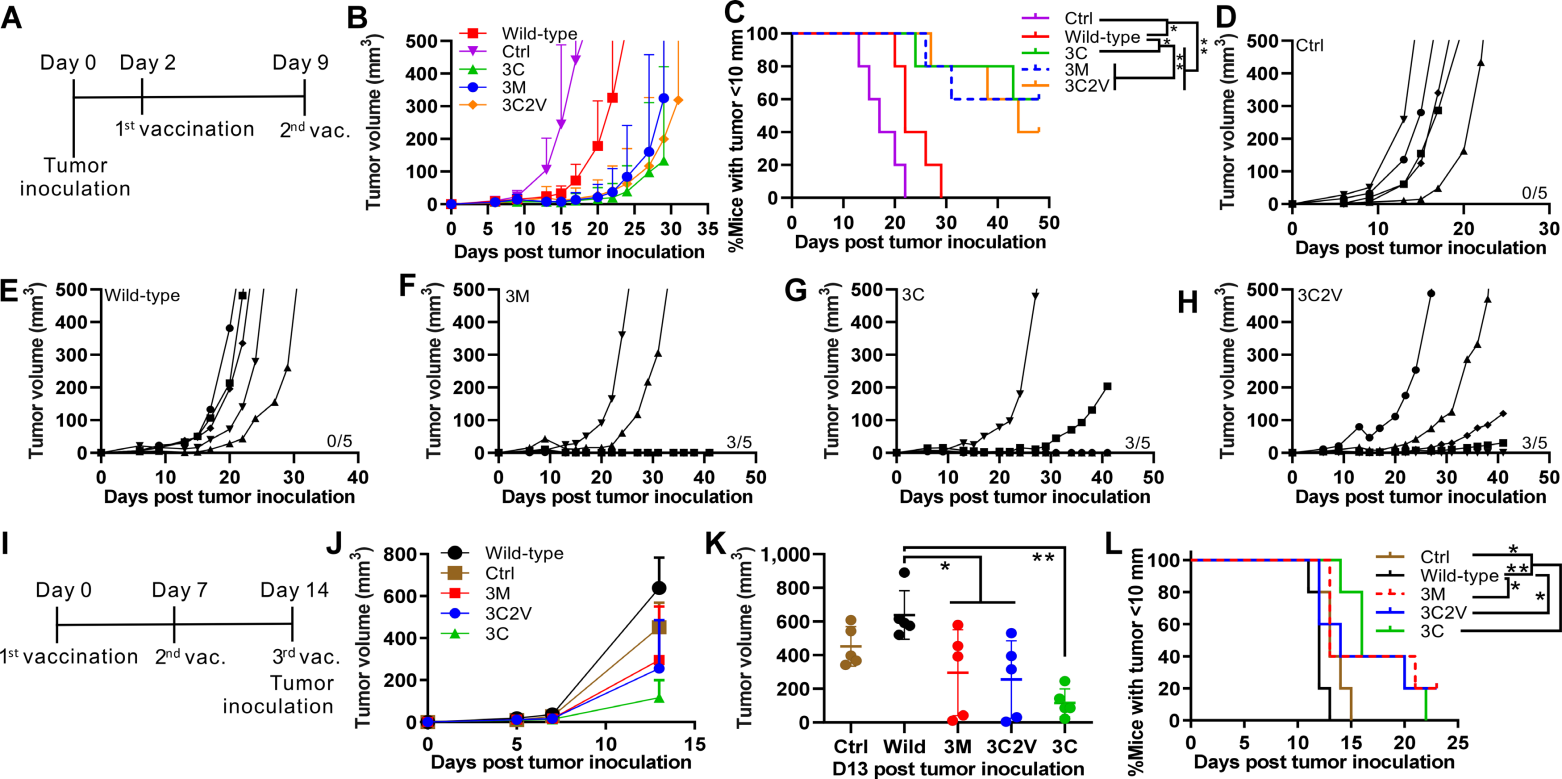
p15E e-mimotope vaccines inhibit tumor growth after immunization. **A,** Schedule of vaccination and tumor inoculation for MC38 therapeutic challenge. Mice were inoculated with 1 × 10^5^ MC38 cells subcutaneously followed with immunization with 2 µg peptide loaded liposomal vaccine on days 2 and 9. Tumor volume (**B**) and the percentage of mice with a tumor diameter <10 mm (**C**). Tumor volume of individual mice from the control group (**D**), wild-type group (**E**), 3M group (**F**), 3C group (**G**), and 3C2V group (**H**). **I,** Schedule of vaccination and tumor inoculation for B16-F10 prophylactic challenge. **J**, Tumor volume of all mice. **K**, Tumor volume on day 13 after tumor inoculation. **L**, The percentage of mice with a tumor diameter < 10 mm. Datapoints represent mean values of 5 mice from the same group for B and J or individual values of 5 mice from the same group in K. Statistical analysis of K applied one-way ANOVA followed by Tukey multiple comparisons tests. Statistical analysis of C and L applied log-rank (Mantel–Cox) test. * and ** indicating *P* < 0.05 and 0.01, respectively. Error bars show mean + or ± SD.

Mice were inoculated with 1 × 10^5^ MC38 cells on day 0, followed by liposomal vaccine immunization on days 2 and 9 with a dose of 2 µg of peptide per mouse per injection, as shown in Fig. 7A. Tumor volume (Fig. 7B) and the percentage of mice with tumor diameters <10 mm (Fig. 7C) indicated significantly reduced MC38 tumor growth following immunization. As expected, no mice in the control or wild-type groups experienced tumor growth inhibition (Fig. 7D and E). All groups immunized with the mimotope vaccines had three mice that showed either no detectable tumor growth or significant tumor growth inhibition by day 42 after tumor inoculation (Fig. 7F–H). The 3C2V mimotope, however, did not show better tumor growth-inhibiting efficacy than the 3M or 3C mimotope in these conditions.

B16-F10 is another p15E-expressing tumor cell line. Mice were immunized three times at 7-day intervals and inoculated with 1 × 10^5^ B16-F10 cells on the same day as the third vaccination as shown in Fig. 7I. Although no mice were cured from the B16-F10 tumor challenge in this prevention setting, the 3C group showed the slowest tumor growth rate compared with all other groups, including the 3C2V group (Fig. 7J). Tumor volumes on day 13 after tumor inoculation in all groups were compared. The wild-type group showed the highest average tumor volume and was significantly different from the 3M, 3C2V, and 3C groups (Fig. 7K). Similarly, the percentage of mice with a tumor diameter <10 mm from all mimotope groups showed significantly reduced tumor growth compared with the wild-type group. No significant differences were observed among the mimotope groups (Fig. 7L).

### TCR Sequencing of Ag-specific T Cells

**FIGURE 8:**
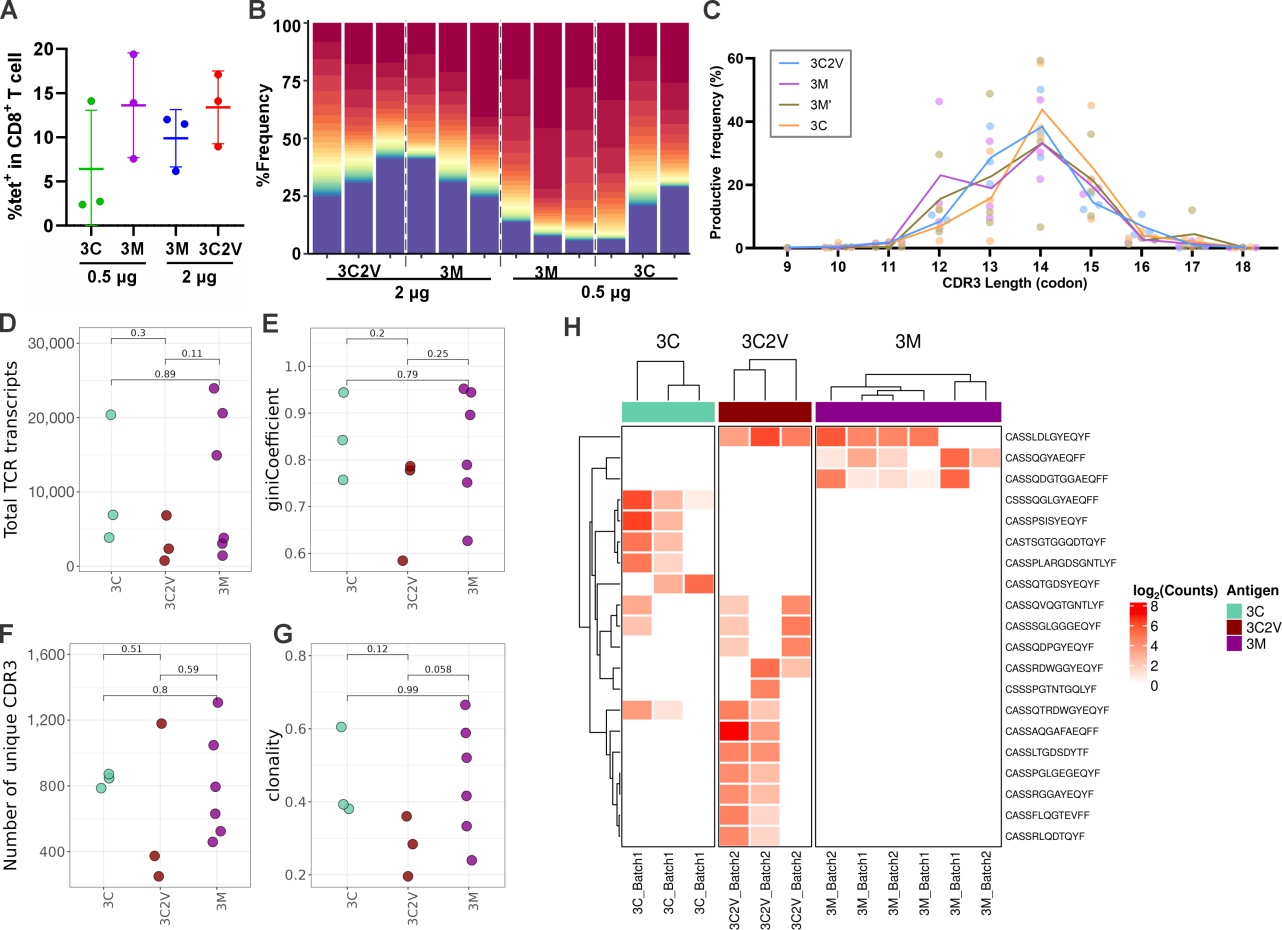
TCR sequencing of wild-type p15E-specific CD8 T cells sorted from splenocytes of 3C, 3M, and 3C2V immunized mice. **A,** Percentage of Ag-specific CD8^+^ T cells in all CD8^+^ T cells. **B,** Proportion of total repertoire occupied by top 30 most abundance clonotypes. The peptide vaccine dose (in µg) used to induce Ag-specific T cells is shown below the graph. **C,** Length distribution of observed CDR3 amino acid sequences within each sample. Lines represent mean proportion within each group. Total TCR transcripts (**D**), Gini coefficient (**E**), number of unique CDR3 clonotypes (**F**), and clonality estimates (**G**) of Ag-specific repertoires. **H**, Differentially abundant clonotypes observed between groups. Datapoints represent individual values for each mouse. Statistical comparisons are determined by Student *t* test. Error bars show mean ± SD.

Mice were immunized twice with the indicated doses of mimotope liposomal vaccine at 7-day intervals. Splenocytes were collected 7 days after the second immunization and stained with p15E wild-type peptide-specific PE-tetramer and anti-CD8 T-cell antibody. The percentage of tetramer-specific CD8^+^ T cells among all CD8^+^ T cells is shown in Fig. 8A. Tetramer-specific CD8^+^ T cells were sorted and collected for DNA extraction before TCR sequencing. The representative gating strategy is shown in Supplementary Fig. S1B and S1C. All the three mimotope liposomal vaccines induced Ag-specific CD8^+^ TCR repertoires that were dominated by the top 30 most abundant clonotypes (Fig. 8B). The general TCRβ repertoire characteristics were similar among Ag-specific T-cell populations. For instance, no significant differences in the CDR3 length distribution (Fig. 8C), the overall TCRβ repertoire transcripts (Fig. 8D), Gini index (Fig. 8E), number of unique clonotypes (Fig. 8F), or clonality (Fig. 8G) were observed among the mimotope groups, suggesting similar clonal characteristics of T cells exposed to various mimotopes. The shared clonotypes of 3C and 3C2V groups indicated a commonality in the antigen structure (Fig. 8H). The 3M group showed more unique clonotypes that were present or not as dominant in 3C and 3C2V. Differential abundance analysis revealed the appearance of several unique clonotypes corresponding to specific mimotope exposures, indicating that while clonal features of responses were similar, specific mimotope peptide structures did impact the TCR landscape.

## Discussion

In this study, we used functional screening strategies to discover three e-mimotopes (p15E-3C, p15E-3M, and p15E-3C2V) that better inhibited p15E-expressing murine tumors with vaccination and better induced CD8^+^ T cells that recognize the native p15E epitope than using the p15E epitope itself as an immunogen. Although p15E is expressed in multiple murine cancer cell lines and is commonly used as an immunologic marker for tumor rejection, it has rarely been successfully used in vaccination studies.

These results validate and expand our recently reported approach to functional screening, which we used to develop an e-mimotope against the Trp2 murine melanoma epitope (10). In this study, we initially identified two new e-mimotopes for p15E by using positional library screening to identify candidate mutations. Next, we identified e-mimotopes with higher reactivity than that of the highly functional but poorly immunogenic p15E-3C e-mimotope by introducing and testing a second mutation based on the predicted MHC-I binding affinity. The identified 3C2V e-mimotope was more immunogenic than the initial p15E-3C mimotope and functional than wild-type p15E. However, despite superior p15E immunogenicity, p15E-3C2V was not more functional than p15E-3C in these experimental conditions. Our screening made use of tumor challenge as the primary marker of e-mimotope activity, which was justified by the observation that mimotopes that induce high-frequency CD8^+^ T cells cross-reactive with the native epitope based on tetramer or IFNγ readouts may not optimally recognize the native epitope in the context of MHC-I expressed on cancer cells. Indeed, this study reveals an additional layer of complexity, in which mimotopes that are sufficiently immunogenic for inducing tumor rejection in a prophylactic challenge should also be tested in a more challenging therapeutic challenge model. Immunogenicity readouts are not a substitute for functional testing.

p15E-3C, p15E-3M, and p15E-3C2V e-mimotopes were superior to the wild-type peptide for inducing cytotoxic CD8^+^ T cells reactive against the wild-type p15E epitope and inhibiting prophylactic MC38 or B16-F10 tumor growth. In therapeutic challenges, these immunogenicity-improved mimotope vaccines were found unable to fully reject tumor growth for all vaccinated mice. This could relate to the local tumor immunosuppression in the therapeutic challenge. It is likely that CD8^+^ T-cell exhaustion in the tumor microenvironment contributed to incomplete responses of the e-mimotopes. By increasing the e-mimotope dose from 0.5 (Fig. 4C) to 2 µg (Fig. 7F), the p15E-3M mimotope showed improved therapeutic effects in the MC38 model. The dose of mimotope Ag could contribute to the antitumor activities, but we did not go further to investigate the dose response for these mimotopes in prophylactic and therapeutic settings. In general, cancer therapeutics are tested in late-stage patients; however, in some cases cancer vaccines have shown promise in a neoadjuvant setting. For example, in a clinical study, neoantigen-specific effector CD8^+^ T cells are related to delayed pancreatic ductal adenocarcinoma recurrence in patients (32).

Throughout these studies, the p15E-3C mimotope exhibited lower immunogenicity than the p15E-3M and p15E-3C2V mimotopes yet showed non-inferior efficacy in therapeutic tumor challenges. The lower immunogenicity of p15E-3C might be explained by the lower predicted MHC-I binding affinity, which was the main motivating factor for screening additional mimotopes that incorporated the 3C mutation. The enhanced function of p15E-3C is presumed to be related to the induction of Ag-specific CD8^+^ T cells that have superior engagement with the TCR-pMHC complex (33). Although p15E-3C2V induced a higher percentage of CD8^+^ T cells reactive against the wild-type p15E sequence compared with the p15E-3C mimotope, no improvement in inhibiting MC38 tumor growth was observed. This is potentially due to the second mutation resulting in induced CD8^+^ T cells that are slightly penalized in stably engaging the p15E bound to MHC-I on cancer cells, compared with T cells induced by the better matching p15E-3C e-mimotope. Nevertheless, further study is required to determine which of these e-mimotopes is the most effective. Short MHC-I–restricted mimotopes have been shown to be tolerant to multiple sequence mutations, which is remarkable considering that this corresponds to a high percentage of the overall sequence (34). MHC-I binding affinity is not the only factor that determines peptide immunogenicity, and TCR-p-MHC affinity is a driving factor that cannot be predicted from the TCR affinity (35). TCR sequencing results showed that the TCR repertoires of 3M and 3C were distinct, but not substantially different in terms of clonality.

The CPQ liposome system, based on the inclusion of CoPoP, PHAD, and QS-21 in liposomes, was instrumental in this study, and we have previously shown that without the CoPoP component, no Ag-specific CD8^+^ T cells are induced (16, 36). In general, the p15E-3C e-mimotope, which has detectable but modest immunogenicity, along with the double mutant p15E-3C2V are candidate epitopes for assessing further improvements to the vaccine adjuvant system, be it through dosage, immunization regime, or testing of additional adjuvants. Furthermore, p15E mimotopes could be assessed in combination with ICB to further elucidate how they could be used in antitumor treatment regimes, and ICB has previously been shown to synergize with CPQ peptide cancer vaccines (10, 16). Finally, future studies could assess how p15E e-mimotopes operate in combination with additional cancer epitopes in a multiplexed immunization scheme.

## Conclusion

The established p15E murine tumor rejection MHC-I epitope was found to be non-functional with immunization using the synthetic peptide and the potent CPQ nanoliposomal vaccine adjuvant system. Multistep functional screening of peptide libraries identified p15E-3C and p15E-3M e-mimotopes, which led to improved immunogenicity against the wild-type Ag and induced antitumor vaccine efficacy. An additional screening step identified a p15E-3C2V mimotope with two mutations, with improved immunogenicity relative to p15E-3C. This study provides considerations for the future design of e-mimotopes and puts forward new e-mimotope candidates for use in preclinical cancer vaccine testing.

## Supplementary Material

Figure S1 Flow cytometry cell gating strategy

Figure S2 CD8+ or CD4+ T cell depletion with murine anti-CD8 or anti-CD4 monoclonal antibodies

Figure S3 p15E-3C2V immunized mice showed long-term resistance to MC38 tumor re-challenge
