# Supplementary material for: Identification of Enhanced Vaccine Mimotopes for the p15E Murine Cancer Antigen
Source: Cancer Res Commun. 2024 Apr 2;4(4):958–69. doi: 10.1158/2767-9764.CRC-23-0384 (PMC10986479; doi:10.1158/2767-9764.CRC-23-0384)
Supplement: Figure S1 — Flow cytometry cell gating strategy [file crc-23-0384-s01.pdf]

Supplementary Figure 1
Identification of Enhanced Vaccine Mimotopes for the p15E Murine Cancer Antigen

Shiqi Zhou[1], Yiting Song[1], Yuan Luo[1], Breandan Quinn[1], Yang Jiao[1], Mark D. Long[2], Scott I. Abrams[3], Jonathan F. Lovell[1*]

[1.] Department of Biomedical Engineering, State University of New York at Buffalo, Buffalo, NY 14260, USA

[2.] Department of Biostatistics & Bioinformatics, Roswell Park Comprehensive Cancer Center, Buffalo, NY 14263, USA

[3] Department of Immunology, Roswell Park Comprehensive Cancer Center Roswell Park Comprehensive Cancer Center, Buffalo, NY 14263, USA

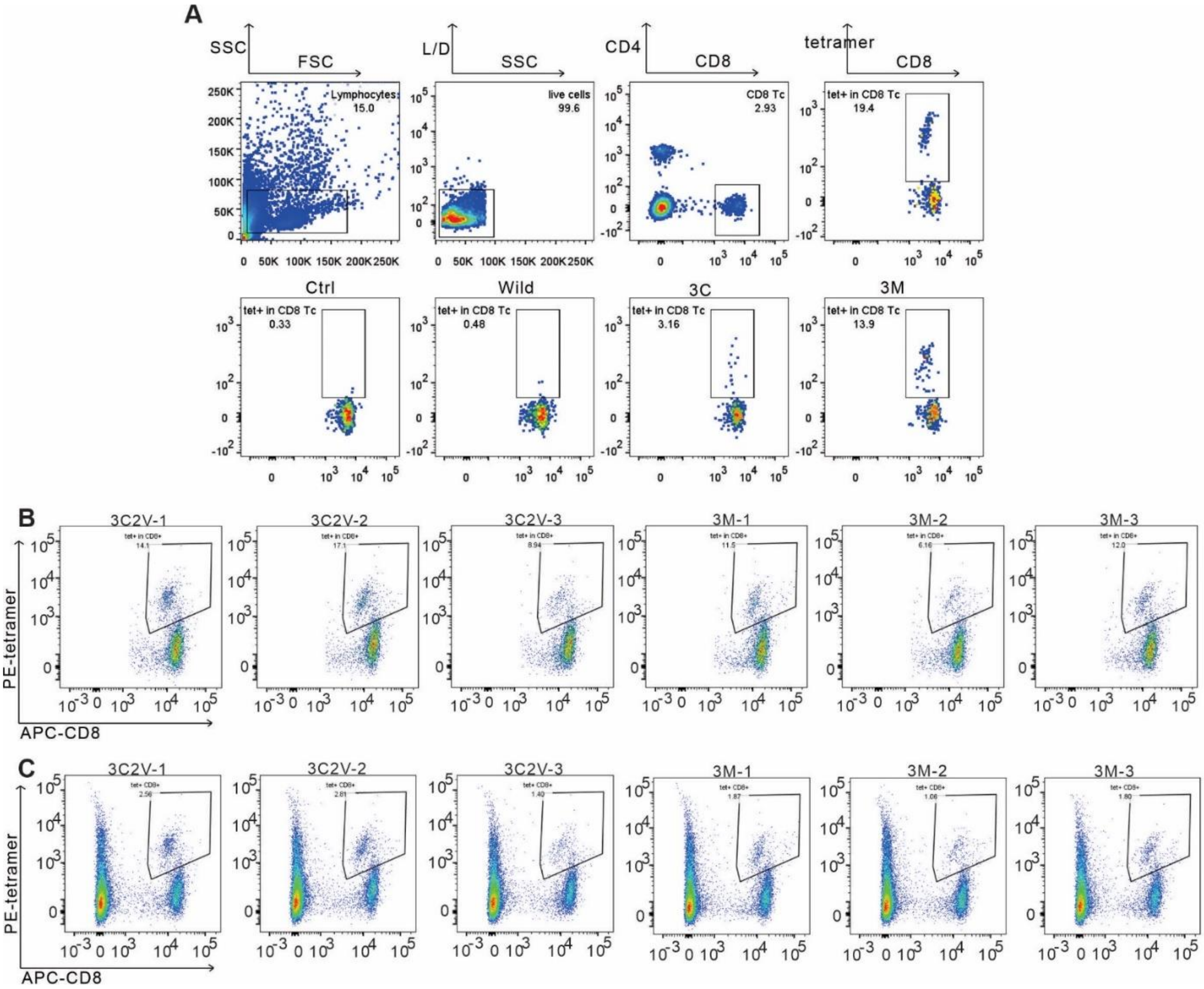

Supplementary Figure S1. Flow cytometry cell gating strategy for **A)** targeting p15E wild type peptide antigen-specific CD8 T cell; Flow cytometry cell gating strategy for sorted **B)** antigen-specific and CD8 T cell-specific double positive cell percentage in all cells and **C)** percentage of antigen-specific cell in all CD 8 T cells.
