# Supplementary material for: Identification of Enhanced Vaccine Mimotopes for the p15E Murine Cancer Antigen
Source: Cancer Res Commun. 2024 Apr 2;4(4):958–69. doi: 10.1158/2767-9764.CRC-23-0384 (PMC10986479; doi:10.1158/2767-9764.CRC-23-0384)
Supplement: Figure S2 — CD8+ or CD4+ T cell depletion with murine anti-CD8 or anti-CD4 monoclonal antibodies [file crc-23-0384-s02.pdf]

Supplementary Figure 2
Identification of Enhanced Vaccine Mimotopes for the p15E Murine Cancer Antigen

Shiqi Zhou[1], Yiting Song[1], Yuan Luo[1], Breandan Quinn[1], Yang Jiao[1], Mark D. Long[2], Scott I. Abrams[3], Jonathan F. Lovell[1*]

[1.] Department of Biomedical Engineering, State University of New York at Buffalo, Buffalo, NY 14260, USA

[2.] Department of Biostatistics & Bioinformatics, Roswell Park Comprehensive Cancer Center, Buffalo, NY 14263, USA

[3] Department of Immunology, Roswell Park Comprehensive Cancer Center Roswell Park Comprehensive Cancer Center, Buffalo, NY 14263, USA

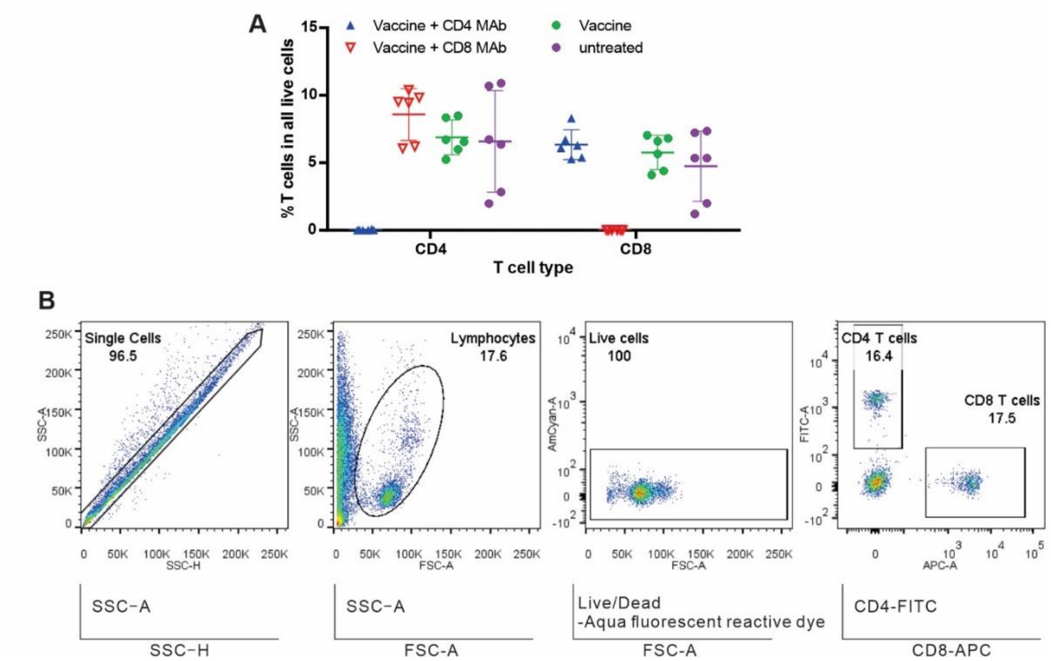

Supplementary Figure S2. CD8+ or CD4+ T cells depletion with murine anti-CD8 or anti-CD4 monoclonal antibodies. Mice received intramuscular injections of indicated vaccine on days 0 and 7 before receiving intraperitoneal injections of 400 µg *InVivo*MAb anti-mouse CD8α antibody or 400 µg *InVivo*MAb anti-mouse CD4 antibody on day 12 and day 13. Mice blood was collected and stained for flow cytometry. **A)** the percentage of $CD4^+$ or $CD8^+$ T cells in all living cells; **B)** the gating strategy of a representative sample from the vaccine-only group.
