# Supplementary material for: Identification of Enhanced Vaccine Mimotopes for the p15E Murine Cancer Antigen
Source: Cancer Res Commun. 2024 Apr 2;4(4):958–69. doi: 10.1158/2767-9764.CRC-23-0384 (PMC10986479; doi:10.1158/2767-9764.CRC-23-0384)
Supplement: Figure S3 — p15E-3C2V immunized mice showed long-term resistance to MC38 tumor re-challenge [file crc-23-0384-s03.pdf]

Supplementary Figure 3
Identification of Enhanced Vaccine Mimotopes for the p15E Murine Cancer Antigen

Shiqi Zhou[1], Yiting Song[1], Yuan Luo[1], Breandan Quinn[1], Yang Jiao[1], Mark D. Long[2], Scott I. Abrams[3], Jonathan F. Lovell[1*]

[1.] Department of Biomedical Engineering, State University of New York at Buffalo, Buffalo, NY 14260, USA
[2.] Department of Biostatistics & Bioinformatics, Roswell Park Comprehensive Cancer Center, Buffalo, NY 14263, USA
[3] Department of Immunology, Roswell Park Comprehensive Cancer Center Roswell Park Comprehensive Cancer Center, Buffalo, NY 14263, USA

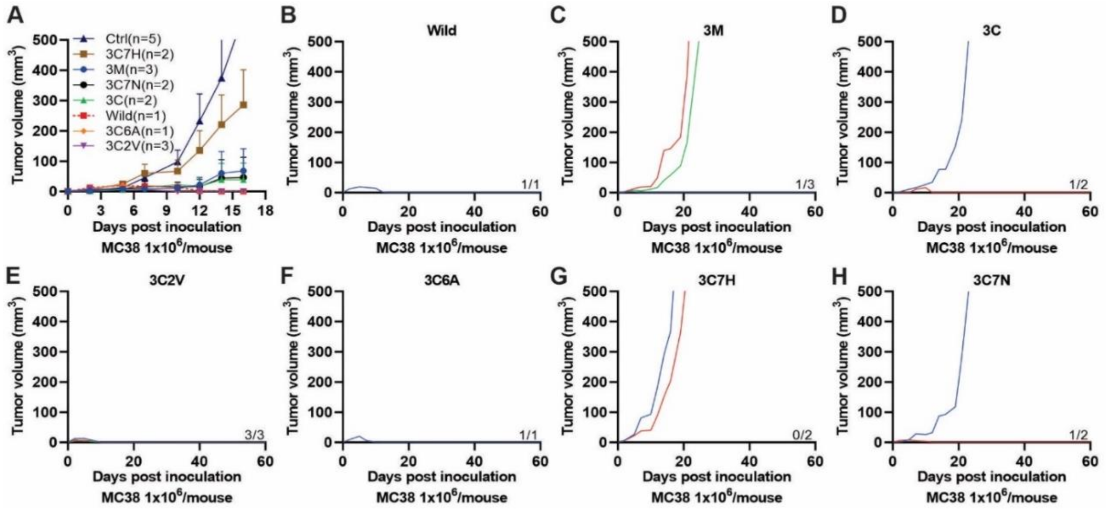

Supplementary Figure S3. p15E-3C2V immunized mice showed long-term resistance to MC38 tumor (re)challenge. One hundred days after the initial MC38 tumor challenge shown in Figure 5, mice that survived the 3C2V peptide vaccine and 5 new Ctrl mice were inoculated with $1x10^6$ MC38 cells. Tumor volume of **A)** all mice; **B)** one mouse from the wild-type group immunized mouse; **C)** three mice from the 3M group; **D)** two mice from the 3C group; **E)** three mice from the 3C2V group; **F)** one mouse from the 3C6A group; **G)** two mice from the 3C7H group; and **H)** two mice from the 3C7N group. The number above the x-axis indicates the number of tumor-free mice out of all mice from the same group.
